# Tuberculosis Infectiousness is Associated with Distinct Clinical and Inflammatory Profiles

**DOI:** 10.21203/rs.3.rs-3722244/v1

**Published:** 2024-01-10

**Authors:** David Horne, Videlis Nduba, Lilian Njagi, Wilfred Murithi, Zipporah Mwongera, Gisella Logioia, Glenna Peterson, R Max Segnitz, Kevin Fennelly, Thomas Hawn

**Affiliations:** University of Washington; Centre for Respiratory Diseases Research, Kenya Medical Research Institute; Centre for Respiratory Diseases Research, Kenya Medical Research Institute; Centre for Respiratory Diseases Research, Kenya Medical Research Institute; Centre for Respiratory Diseases Research, Kenya Medical Research Institute; University of Washington; University of Washington; University of Washington; National Heart, Lung, and Blood Institute (NHLBI), National Institutes of Health (NIH); University of Washington

## Abstract

Interrupting transmission events to prevent new acquisition of infection and disease is a critical part of tuberculosis (TB) control efforts. However, knowledge gaps in understanding the biology and determinants of TB transmission, including poor estimates of individual infectiousness and the lack of accurate and convenient biomarkers, undermine efforts to develop interventions. Cough-generated aerosol cultures have been found to predict TB transmission better than any microbiological or clinical markers in cohorts from Uganda and Brazil. We hypothesized that highly infectious individuals with pulmonary TB (defined as positive for cough aerosol cultures) have elevated inflammatory markers and unique transcriptional profiles compared to less infectious individuals (negative for cough aerosol cultures). We performed a prospective, longitudinal study using a cough aerosol sampling system as in other studies. We enrolled 142 participants with treatment-naïve pulmonary TB in Nairobi, Kenya, and assessed the association of clinical, microbiologic, and immunologic characteristics with Mtb aerosolization and transmission in 143 household members. Contacts of the forty-three aerosol culture-positive participants (30%) were more likely to have a positive IGRA (85% vs 53%, P = 0.005) and a higher median IGRA IFNγ level (P < 0.001, median 4.25 IU/ml (0.90–5.91) vs. 0.71 (0.01–3.56)) compared to aerosol culture-negative individuals. We found that higher bacillary burden, younger age, and larger mean upper arm circumference were associated with positive aerosol cultures. In addition, novel host inflammatory profiles, including elevated serum C-reactive protein and sputum cytokines, were associated with aerosol culture status. Notably, we found pre-treatment whole blood transcriptional profiles associated with aerosol culture status, independent of bacillary load. Together, these findings suggest that TB infectiousness is associated with epidemiologic characteristics and inflammatory signatures and that these features may be used to identify highly infectious persons. These results provide new public health tools and insights into TB pathogenesis.

## INTRODUCTION

Tuberculosis (TB), a leading infectious disease-related cause of death,(1) is spread person-to-person through the inhalation of aerosolized bacilli. A key step for TB elimination is interrupting transmission events to prevent new acquisition of infection and disease. However, knowledge gaps in understanding the biology and determinants of TB transmission undermine efforts to develop interventions.(2, 3) These gaps include uncertainties around the determinants of infectiousness, poor estimations of individual infectiousness, and the lack of accurate and convenient biomarkers of infectiousness. Similar to other infectious diseases,(4–6) “superspreaders”, individuals with TB who are responsible for the majority of *Mycobacterium tuberculosis (*Mtb) transmission,(7–11) may have an outsized role in TB endemic settings. Modeling studies suggest greatly amplified returns by focusing control efforts on the minority of persons with TB who are most infectious.(12) Although the existence of “superspreaders” in Mtb transmission remains unproven, investigations into this phenotype may provide insights into TB pathophysiology and inform the development, implementation, and evaluation of targeted public health interventions to eliminate TB.(3)

Epidemiologic studies of Mtb infectiousness suggest that several host characteristics, including younger age, higher bacillary load, cough features, and greater contact time are associated with increased transmission.(13–18) Overall, these associations are weak and suggest that additional factors regulate infectivity. Studies by Wells and Riley from the pre-chemotherapeutic era demonstrated that a subset of patients with TB generated small droplets capable of infecting guinea pigs.(15, 19–21) More recent studies focused on people living with HIV (PLWH) similarly found that a subset of patients transmitted Mtb.(22) Compared to sputum smear and culture assessment, direct measurement of Mtb aerosolization with a cough aerosol sampling system (CASS)(23–28) is a more accurate predictor of transmission among household contacts (HHCs).(24, 26) A recent study suggested that sputum bacillary load and clinical characteristics, including fewer symptoms and a stronger cough, predict Mtb aerosolization.(29) Studies using a Respiratory Aerosol Sampling Chamber (RASC), which directly measures viable Mtb in bioaerosols,(30–32) as well as those that used face mask sampling(33, 34) suggested that tidal breathing and non-cough mechanisms may be important drivers of transmission.(35) Although these studies demonstrate features of airborne Mtb transmission, the biologic mechanisms remain poorly understood, including whether immune pathways modulate aerosolization. In addition, while CASS and RASC are important research tools, a simple diagnostic test or algorithm that identifies superspreaders has not been developed and would have significant impact on efficient resource allocation for TB control. (36, 37)

To address these gaps in knowledge, we designed the TB Aerobiology, Immunology and Transmission (TBAIT) study. We performed a prospective, longitudinal study using CASS, enrolled 142 participants with pulmonary TB, and assessed the association of clinical, microbiologic, and immunologic characteristics with Mtb aerosolization. In addition, we measured IGRA responses in HHCs to assess Mtb transmission risk by cough aerosol culture status of the index participant.

## METHODS

### Study Design

#### Study Setting & Participants

Between March 1, 2021, and March 30, 2023, we enrolled adults with newly diagnosed pulmonary TB in Nairobi, Kenya. The TB incidence rate in Kenya was estimated to be 558 per 100,000 adults in 2015.(38) Participants were enrolled either through outpatient TB and respiratory clinics where they had presented for healthcare (passive case finding) or were diagnosed with pulmonary TB though a Nairobi-based household TB prevalence survey (active case finding). Participants identified through active case finding underwent chest x-ray (regardless of symptoms) and cough assessment; those with an abnormal chest x-ray and/or who reported a current cough were asked to provide two sputum samples for AFB-smear, - culture, and GeneXpert testing. All participants were diagnosed with TB based on a positive GeneXpert test result, either GeneXpert MTB/RIF (Xpert MTB/RIF) or GeneXpert Ultra (Xpert Ultra). AFB-culture was subsequently performed on sputum samples. A final diagnosis of pulmonary TB was defined as a positive GeneXpert test unless the result was “trace positive” from the Xpert Ultra assay, in which case culture confirmation of Mtb was required. Participants had not initiated anti-TB treatment at the time of enrollment and study interventions. Potential participants who were unable to consent in the study languages (Ki-Swahili, English), did not provide a home location, planned to move from the area within six months, declined study procedures, or were currently imprisoned were not eligible for the study.

We also enrolled the household contacts of participants with pulmonary TB to assess for evidence of TB transmission events. Household contacts were eligible for enrollment if they resided and slept in the household for at least 60 days prior to enrollment. There were no age restrictions on the eligibility of household contacts.

#### Ethical Approvals

This study was approved by the Kenya Medical Research Institute Scientific and Ethics Review Unit (048/3988) and the University of Washington Institutional Review Board (STUDY00009209).

### Study Procedures

#### Participants with TB:

We collected sputa from participants at enrollment (“spot”) and the following morning (“morning”). AFB-smear and culture were performed on both samples, and GeneXpert testing was performed on the spot sample. We collected a separate sputum for cytokine testing in addition to whole blood for laboratory testing and mRNA analysis.

CASS consists of a six-stage Andersen Cascade Impactor (Thermo Fischer Scientific, Rockford, IL) within a larger stainless-steel chamber attached to tubing and with a vacuum pump creating airflow through the system.(23, 25) We calibrated the vacuum pump flow rate (28.3 L/min) using a primary flow calibrator (Model 4046, TSI, Inc., Shoreview, MN), and then maintained and monitored that using a marked field rotameter (SKC, Inc., Eighty Four, PA). Each of the six stages of the Andersen cascade impactor holds a Middlebrook 7H10 or 7H11 solid agar plate, on which aerosolized particles impact based on particle size. The agar plates were loaded into the Andersen impactors in sterile fashion; the impactors were loaded into the cylinder before each study. Prior to cough peak flow measurements and sputum collection, participants coughed into a mouthpiece on tubing connected to the chamber for 5 minutes. Plates were incubated at 37°C for 8 weeks and observed weekly for growth. When growth was identified as Mtb, colony forming units (CFU) were counted. Agar plates with non-acid fast bacilli growth were considered contaminated and discarded. The cylinder and components were autoclaved after each use. Mtb growth was first detected at a median of 4 weeks (IQR, 4–6) and was most frequently detected on plates four (22 with growth), five (29), and six (19), corresponding to particle sizes of 2.1–3.3 μm, 1.1–2.0 μm,, and 0.65–1.1 μm, respectively.(23) (Supplemental Fig. 1) Among cough aerosol culture participants, the maximal plate CFUs, defined for each participant as the highest CFU count for any plate with Mtb growth, ranged from 1 to 76 with a median of 12 CFUs (IQR, 4–27).

For cough peak flow (CPF) measurements, we instructed participants to cough as forcefully as possible through a disposable mouthpiece attached to a Vitalograph peak flow meter (Ennis, Ireland). The procedure was performed three times and results recorded in L/min. The highest recorded value was used in analyses. Enrollment posteroanterior (PA) chest x-rays were obtained and interpreted by a member of the study team (DJH, a pulmonologist) for the presence of cavitations and number of quadrants with changes attributed to TB disease. Study personnel interviewed participants to collect demographic information, HIV history, and TB history. To assess cough-related effects on quality of life, we administered the Leicester Cough Questionnaire (LCQ), a validated health status measure for adults with chronic cough ranging from 3–21 with a lower score indicating greater impairment.(39)

#### Household contacts:

Consenting household contacts were interviewed to assess for symptoms consistent with TB and to collect demographic information and exposure history. Household contacts underwent chest x-ray (PA unless age < 10 years, in which case PA and lateral images were obtained) and phlebotomy for interferon-gamma release assay and HIV testing. Household contacts who were suspected of having TB based on an abnormal chest x-ray and/or the presence of symptoms (cough, fever, weight loss) had sputum collected for GeneXpert testing.

### Laboratory assays

Sputum volume and quality were visually assessed by laboratory personnel. We performed concentrated sputum smears on all samples using Auramine O staining with fluorescence smear microscopy, read according to WHO AFB scale. Sputum was processed using the NALC-NaOH-NaCitrate method and 0.5 ml of the pellet inoculated in mycobacterial culture (MGIT) Mycobacterium Growth Indicator 4 mL tubes (Becton-Dickinson, Franklin Lakes, NJ) supplemented with BD BBL^™^ MGIT^™^ PANTA and incubated in an automated BACTEC MGIT 960 machine for growth determination. Samples that had zero (0) growth units at 42 days were confirmed negative. Broth cultures that were flagged as positive by the MGIT 960 had the time to detection recorded and the presence of acid-fast bacilli verified using Ziehl-Neelsen smear microscopy with isolates identified as Mtb using the MGIT TBc Identification Test (Becton-Dickinson Diagnostic Instrument Systems, Sparks, MD). Further speciation was not performed. Xpert MTB/RIF or Xpert Ultra (Cepheid, Sunnyvale, CA) were performed on raw sputum samples. GeneXpert results were recorded as a cycle threshold (Ct) value and as a semi-quantitative grade: Negative, Very Low, Low, Medium, High, with an additional category of “Trace” for Xpert Ultra. Results from GeneXpert testing for rifampin resistance were recorded as negative, positive, or indeterminate. For the CASS culture plates, after the preparation and autoclaving of 7H10 or 7H11 solid media, appropriate volumes of OADC (Oleic acid Albumin Dextrose Catalase) and antibiotics (amphotericin B, carbenicillin, polymixin B and trimethoprim lactate) were added to inhibit contaminant growth.

C-reactive protein (CRP) was measured from serum samples using Cobas C 111 chemistry analyzer (Roche Diagnostics Ltd, Liechtenstein, Switzerland) according to the manufacturer’s instructions. The detection range was 0.6–350 mg/L. We offered HIV testing to participants unless a participant was known to be living with HIV or if testing had been performed within the six months prior to enrollment. Whole blood for QuantiFERON-TB Plus testing (QFT-Plus, Qiagen Diagnostics; Hamburg, Germany) was drawn into lithium heparin blood collection tubes, and 1 mL amounts were transferred into Nil, Mitogen, TB1, and TB2 tubes. All QFT-Plus tubes were incubated at 37°C within 6 hours of collection. QFT-Plus processing and interpretation was performed according to the manufacturer’s instructions.(40) If TB1-nil and/or TB2-nil were > 0.35 IU/ml and > 25% of nil value (with Nil < 8.0 IU/ml), then the QFT-Plus test was considered positive. If Nil > 8.0 IU/mL or Mitogen–Nil < 0.5 IU/ml with Nil < 8.0 IU/ml and negative antigen-Nil results, then QFT-Plus was defined as indeterminate. QFT-Plus results were negative if Nil < 8.0 IU/ml with either antigen-Nil values < 0.35 IU/ml IFN-γ or < 25% of Nil). Indeterminate results were repeated, with the second result reported.

### Sputum Cytokines

Spot sputum was digested by adding an equal volume of 10% Sputolysin (Millipore, Merck KGaA, Darmstadt, Germany). The mixture was vortexed and incubated at 37°C for 15 minutes. The digested sample was centrifuged at 500xg for 10 minutes. The supernatant was preserved by the addition of 40μl of 25X cOmplete^™^ protease inhibitor cocktail solution (Roche, Merck KGaA, Darmstadt, Germany) per 1ml of sample and stored at −80°C. Sputum supernatants were tested for CXCL8 (IL-8), Interleukin 1 beta (IL-1β) and Interleukin 6 (1L-6) using sandwich ELISA according to the manufacturer’s instructions. (R&D Systems Inc. Minneapolis, USA.).

### Whole blood transcriptomics: RNA isolation, RNASeq, Data processing and Analysis

PAXgene tubes were thawed at room temperature and RNA was isolated using PAXgene miRNA spin columns (Qiagen), followed by globin reduction using GlobinClear Human (ThermoFisher). To generate sequencing libraries, total RNA (0.5 ng) was added to reaction buffer from the SMART-Seq v4 Ultra Low Input RNA Kit for Sequencing (Takara), and reverse transcription was performed followed by PCR amplification to generate full length amplified cDNA. Sequencing libraries were constructed using the NexteraXT DNA sample preparation kit (Illumina) to generate Illumina-compatible barcoded libraries. Libraries were pooled and quantified using a Qubit^®^ Fluorometer (Life Technologies). Sequencing of pooled libraries was carried out on a NextSeq 2000 sequencer (Illumina) with paired-end 59-base reads, using a NextSeq P3 sequencing kit (Illumina) with a target depth of 5 million reads per sample. Base calls were processed to FASTQs on BaseSpace (Illumina), and a base call quality-trimming step was applied to remove low-confidence base calls from the ends of reads. The FASTQs were aligned to the GRCh38 human reference genome, using STAR v.2.4.2a and gene counts were generated using htseq-count. QC and metrics analysis was performed using the Picard family of tools (v1.134). Counts were assigned to gene exons using RSEM 1.3.0. Further RNA sequencing data filtering and analysis were performed in R 4.2.3.(41) We removed libraries with fewer than 1,000,000 total reads or a median coefficient of variation of coverage (median CV) greater than 0.8; this resulted in the removal of 2 sequencing libraries, both of which were clear outliers by these metrics. Libraries were reduced to protein-coding genes, and principal component analysis (PCA) did not detect any strong outliers based on RNA composition. Protein-coding counts were normalized for using the trimmed mean of M-values normalization method and filtered to protein coding genes with at least 5% of libraries containing at least 1 count per million (CPM). Finally, counts were converted to log2 CPM using the R package “voom”.(42)

## Data Analysis

### Host Characteristics and Household Contact Analyses

Our primary outcome of interest was cough aerosol culture status. Participants were considered cough aerosol culture-positive if one or more of the six CASS plates were positive for Mtb growth. Participants were considered cough aerosol culture-negative if no CASS plates were positive for Mtb growth and no more than two of the six plates were discarded as contaminated with fungal or bacterial overgrowth. We assessed associations between predictors and outcomes using bivariate and multivariable logistic regression. Multivariable models were developed using forward selection stepwise regression, evaluating variables with p-values ≤ 0.20 in bivariate analyses and retaining covariates which significantly improved model fit based on likelihood-ratio tests. We assessed multicollinearity between our independent variables using variance inflation factors and condition indices. All statistical tests were two-sided with α = 0.05. We assessed associations between cough aerosol culture-positive status and host TB characteristics using bivariate and multivariable logistic regression in which we evaluated the following predictor variables: age, sex, HIV status, body mass index (BMI), mid-upper arm circumference (MUAC), TB symptoms (cough, fever, night sweats, weight loss), cough duration, Leicester Cough Questionnaire (LCQ) score, prior history of TB, cough peak flow, CRP, hemoglobin, white blood cell count, hemoglobin A1C percent, sputum appearance, GeneXpert semi-quantitative grade, GeneXpert Ct values, AFB-smear grade, time to detection (TTD) of Mtb growth in liquid culture, presence of chest X-ray cavitation, and number of radiographic quadrants with TB-related changes. GeneXpert Ct values were assigned using the smallest Ct value from any of the probes targeting the rpoB gene; participants with Xpert Ultra trace positive results (for whom rpoB probe Ct values were 0) were assigned a Ct value of 35, near the highest detectable Ct value for Xpert Ultra.

To evaluate associations between cough aerosol culture status and evidence of TB transmission, we investigated QFT-Plus responses in household contacts by cough aerosol culture status of the index participant. QFT-Plus responses were dichotomized as positive (≥ 0.35 IU/mL) or negative (< 0.35 IU/mL) after excluding indeterminate responses. We also evaluated the absolute interferon-gamma response based on the larger value of either TB antigen 1 – nil or TB antigen 2 – nil. To evaluate associations with QuantiFERON positive results in household contacts, we used bivariate and multivariable logistic regression models with random intercepts (*melogit* command in Stata) to account for clustering by index participant and compared multivariable models with the likelihood-ratio test. We performed analyses using Stata 14 (StataCorp, College Station, TX) and R: A Language and Environment for Statistical Computing.

### Risk Score Modeling

Analyses were performed to develop a risk score for clinical decision making to identify highly infectious (cough aerosol culture-positive) persons with pulmonary TB. We evaluated covariates from our multivariable logistic regression model that could be applied at the time of a patient’s diagnosis with pulmonary TB. Included variables were dichotomized and the optimal cut-points were determined using Youden index (*J*) method, the point maximizing the Youden function which is the difference between true positive rate and false positive rate over all possible cut-point values.(43) These values were then used in a multivariable logistic regression model. The risk score was generated by dividing the beta coefficients from the logistic regression model by the smallest beta in the model, multiplying by 3, and rounding to the nearest integer. Internal validation was conducted using the bootstrap resampling method with 5000 replications. We evaluated Somers’ D_xy_ index, a rank correlation between predicted probabilities and observed responses, to evaluate model performance.(44) Somers’ D_xy_ index takes values between − 1 and 1, with the latter demonstrating agreement between predicted and observed responses.

### Cytokine Analyses

Association of cytokine concentrations (log10 pg/ml) with cough aerosol culture status and cavitary disease was done by Wilcoxon rank sum test with continuity correction. Bivariate logistic regression models were used to compare cytokine concentration with cough aerosol culture status, GeneXpert cycle thresholds, and number of chest x-ray quadrants with changes attributed to TB disease.

### RNASeq Analysis: Estimation of Differential Expression

Fold changes in gene expression were estimated using linear models implemented in the R package “kimma”.(45). In addition to primary fixed effect of cough aerosol culture, model selection considered for inclusion clinical covariates which significantly associated with cough aerosol culture status in simple bivariate modeling, and which were complete for our RNASeq subset (Supplementary Table 1). We compared single covariate additions of bacillary load (GeneXpert Ct), age, and cavitary disease, assessing trends in model fit using the Akaike Information Criterion (AIC) across the genome (Supplementary Fig. 3A). We then performed stepwise model addition, comparing impact on AIC at each step, and only retained covariates which significantly improved model fit (delta AIC< −2) for at least 10% of genes (Supplementary Fig. 3B). This resulted in an expression model accounting for bacillary load in addition to the cough aerosol culture phenotype (Supplementary Fig. 3C-D). While a strong majority of the genes (78%) were best fit by models including cough aerosol culture status alone, 16% of genes were best fit with the inclusion of bacillary load with a smaller percentage best explained by more complex models with age and cavitary disease (Supplemental Fig. 3A). We used a final model for cough aerosol culture status as the primary outcome with bacillary load as a covariate.

### RNASeq Analysis: Gene Set Enrichment

We used a competitive gene set test to evaluate enrichment of gene expression differences between cough aerosol culture status (positive vs. negative). We used gene set enrichment analysis (GSEA) using the pre-ranked approach employed in the fast GSEA (FGSEA) method and implemented in the R package “fgsea”,(46) and tested for enrichment of the Hallmark annotated biological pathways accessed via the mSigDB database.(47) Log2 Fold change estimates (log2 FC) for cough aerosol culture status, adjusted for the effect of bacillary load, were used to pre-rank genes for GSEA. Estimates for the effects of bacillary load were separately tested for enrichment in GSEA to identify enriched pathways unique to cough aerosol culture status. For each clinical predictor (cough aerosol culture status, bacillary load), we used kimma estimated log2FC to pre-rank genes and used gene-set permutation with 1000 permutations to randomize gene ordering.(45)

## RESULTS

### Cough aerosol culture results among participants with pulmonary TB

To examine the biology of Mtb aerosolization and transmission, we enrolled 142 individuals with microbiologically confirmed pulmonary TB in Nairobi, Kenya. The median age of participants was 35 years (interquartile range (IQR), 27–44) and 27% were women (Table 1). Most participants were symptomatic including cough in 95% and weight loss in 85%. Most participants had medium (32%) or high (39%) GeneXpert semi-quantitative grades and findings of cavitations on chest x-ray (57%). Only 10% of participants were living with HIV, of whom one-half were taking antiretroviral therapy at enrollment. The median CRP value among all participants was high at 67 mg/L. Forty-three participants (30%) were cough aerosol culture-positive and had higher measures of bacillary burden, including higher AFB-smear grades and shorter time to detection of Mtb growth in liquid culture, compared to cough aerosol culture-negative participants (Table 1). Cough aerosol culture-positive participants were more likely to have cavitary lung disease and at least two quadrants affected by TB on chest x-ray than cough aerosol culture-negative persons. GeneXpert cycle threshold and semi-quantitative grade were significantly higher among cough aerosol culture-positive compared to cough aerosol culture-negative participants. More cough aerosol culture-positive participants (91%) had GeneXpert semi-quantitative grades of medium or high compared to 62% of cough aerosol culture-negative individuals. The correlation between GeneXpert cycle threshold and cough aerosol culture status was − 0.44, a moderate correlation which suggests that additional factors determine aerosolization.

### Index cough aerosol culture positivity associated with IGRA positive result in household contacts

We next evaluated IGRA results in household contacts to determine whether cough aerosol culture status is associated with infectiousness. We enrolled 143 household contacts of 55 index participants (Table 2). In bivariate analyses, household contacts did not differ in gender or age by cough aerosol culture status of the index person. The overall frequency of QFT positive results among household contacts was 60% and differed by cough aerosol culture status of the index participant: 85% and 53%, for contacts of cough aerosol culture-positive and -negative participants, respectively (P = 0.005). Cough aerosol culture-positive contacts had a higher median IGRA IFNγ level compared to cough aerosol culture-negative individuals (P < 0.001, median 4.25 IU/ml (0.90–5.91) vs. 0.71 (0.01–3.56)). Among participants who were QFT-positive, the mean IFN-γ levels were significantly higher among contacts of cough aerosol culture-positive persons (n = 22, 4.76, SD 2.58) compared to contacts of cough aerosol culture-negative persons (n = 55, 3.35, SD 2.04, P = 0.01).

Using mixed effects logistic regression models, we next evaluated bivariate associations between measures of index case TB infectiousness and QFT results in contacts. Older age of the household contact was significantly associated with QFT-positive result as were index characteristics of cough aerosol culture positivity, cavitary findings on chest x-ray, and not having HIV infection. (Table 3) Using multivariable models with random intercepts to account for clustering by index participant, we evaluated predictors of interest for associations with QFT positivity in household contacts and found that the best performing model included index cough aerosol culture and HIV status, and household contact age. (Table 3)

### Models of index characteristics associated with cough aerosol culture status

**Figure 1:**
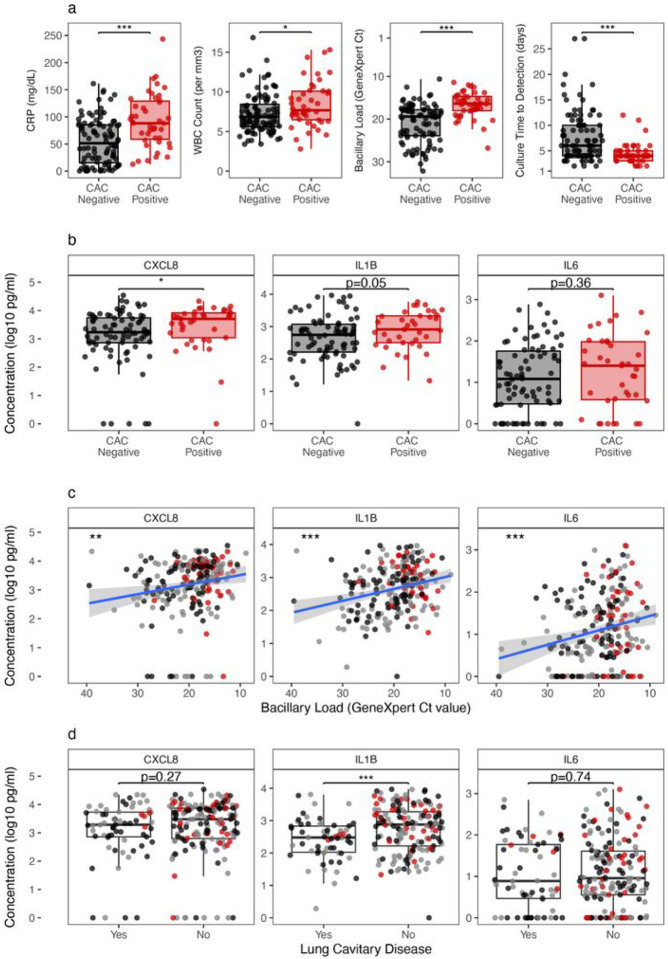
Association of blood and sputum inflammatory and microbiologic markers with cough aerosol culture positivity. Inflammatory and microbiologic markers were measured in blood and sputum at baseline visit and correlated with cough aerosol culture positivity. (a) Serum CRP, Blood WBC, sputum bacillary load determined by GeneXpert Ct value, and sputum culture time to detection. (b-d) Sputum CXCL8, IL1B, and IL6 measurements at diagnosis were correlated with cough aerosol culture positivity (b), bacillary load (c), and chest x-ray lung cavitation (d). For c-d, cough aerosol culture-positive and cough aerosol culture-negative individuals are depicted with red or black circles, respectively. Grey circle, unknown cough aerosol culture status. *p<0.05, **p<0.01, ***p<0.001

We evaluated host characteristics associated with cough aerosol culture status (Table 1). On bivariate analyses, younger age, higher MUAC, cavitary disease on chest X-ray, TB-related abnormalities in more chest X-ray quadrants, and higher cough-related impairment effects on quality of life (lower Leicester Cough Questinnaire score) were associated with cough aerosol culture-positive status. Factors associated with higher bacillary burden including lower GeneXpert Ct value, higher GeneXpert grade, higher sputum smear grade, and shorter time-to-detection of Mtb culture growth were all associated with cough aerosol culture-positive status. Higher WBC count and CRP level were also associated with cough aerosol culture-positive status (Fig. 1A). We compared multivariable models for predictors associated with cough aerosol culture-positive status and found that the best performing model included lower GeneXpert Ct value, lower age, higher CRP level, higher MUAC, and shorter TTD of culture growth (Table 4). The coefficient of determination (R^2^) of this model was 0.38, suggesting that factors in addition to the evaluated independent variables determine TB infectiousness.

#### Performance of clinical prediction rule to identify cough aerosol culture-positive persons.

We developed and evaluated the performance of a clinical prediction rule to predict cough aerosol culture-positive persons as a means to identify those who are likely to be highly infectiousness. We included predictors from our multivariable logistic regression model that are readily available to a clinician during the initial visit. For this reason, we removed time-to-detection of culture growth but kept CRP level as there are point-of-care versions of this test.(48) Using the coefficients obtained in the multivariable logistic regression analysis we derived the following prediction equation for cough aerosol culture -positive status where outcomes were coded according to weighted scores based on the beta coefficient: CRP > 42.9 + Age < 43.8 years + MUAC > 22.8 cm + Xpert Ct < 16.7. A total risk score is calculated by adding the risk points and has an optimum cut point of 11 points for predicting cough aerosol culture-positive status (Supplemental Table 1). The prediction rule based on the calculated risk score had an area under the receiver operating curve (AUROC) of 0.869 (95% CI: 0.807–0.930)(Supplemental Fig. 2). The model’s Somers’ D_xy_ index was 0.73; the equivalent in bootstrap validation was 0.71, with an optimism estimate of 0.032, indicating good stability of the model in internal validation.

#### Sputum cytokines are associated with cough aerosol culture positivity, bacterial load, cavitary lung disease, and extent of lung involvement.

To further examine the association of inflammatory markers with cough aerosol culture -positive status, we examined sputum levels of CXCL8 (IL-8), IL-1β, and IL-6 (Fig. 1B). The concentrations of CXCL8 and IL-1β were significantly higher among the cough aerosol culture-positive compared to cough aerosol culture-negative participants (p-value 0.017 and 0.031, respectively) with IL-6 having no difference (p-value 0.27) (Fig. 1B). In addition, higher IL-6, CXCL8, and IL-1β levels were associated with greater bacillary burden (measured by GeneXpert cycle threshold, p-value 0.0009, 0.007 and 0.00007, respectively, Fig. 1C). IL-1β, but not IL-6 or CXCL8, was positively associated with cavitary lung disease (p-value 0.0007) across cough aerosol culture status. Together, these data suggested that three sputum cytokines were variably associated with cough aerosol culture-positive status, bacterial load, and cavitary lung disease.

#### Whole blood transcriptomic signatures are associated with cough aerosol culture positivity and sputum bacterial load.

**Figure 2:**
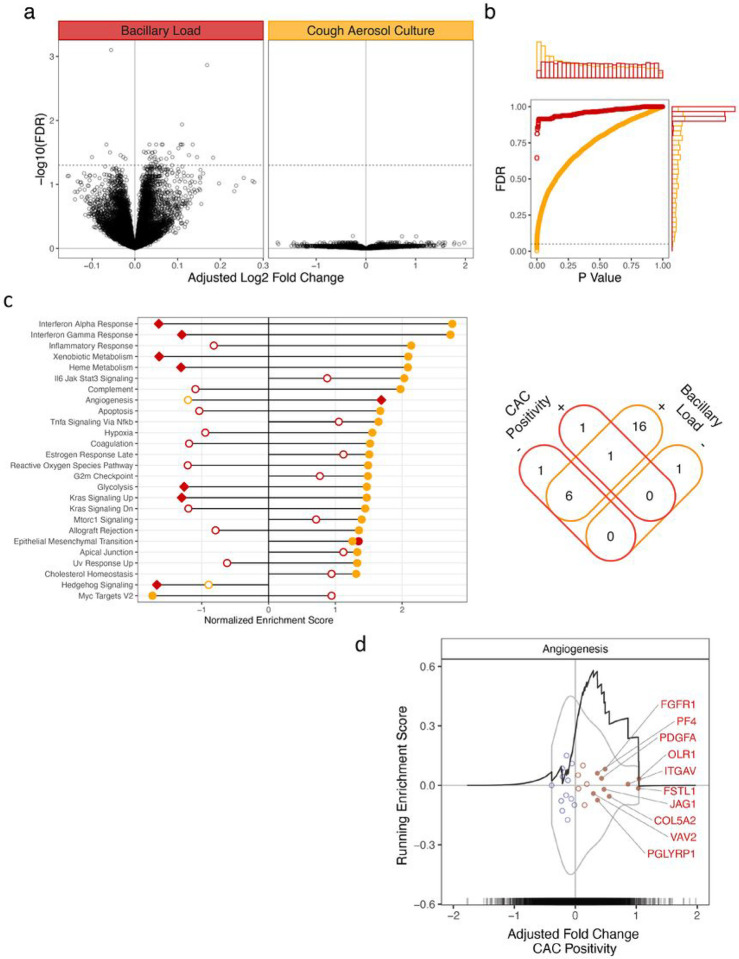
Whole blood transcriptional signatures and association with cough aerosol culture positivity and bacillary burden. a) Differential gene expression associated with cough aerosol culture positivity and bacillary load. Volcano plot shows the gene-wise covariate-adjusted log2 fold change associated with each effect as well as each gene’s Benjamini-Hochberg adjusted false discovery rate (FDR). b) Distributions of unadjusted p-values (x axis) and FDR (y axis) for each effect. c) Gene set enrichment of Hallmark pathways in differential gene expression of cough aerosol culture positivity (red) and bacillary load (yellow). Displayed at left are all pathways for which any effect showed enrichment at an FDR threshold of 0.2. For each effect, pathways with an enrichment FDR<0.2 are indicated with solid points while open points reflect FDR>=0.2; diamond shaped points locate two pathways uniquely enriched in the expression signal associated with cough aerosol culture positivity. Pathways are organized by their maximum enrichment score for any of the two effects. Venn diagram at right displays the number of pathways enriched in each effect at FDR<0.2 with stratification by concordance of directionality of effect. d) Leading edge genes driving enrichment of Hallmark pathways uniquely associated with cough aerosol culture-positive status. Points indicate the adjusted fold change associated with cough aerosol culture positivity (y position is arbitrary jitter), are colored according to sign of FC, and filled/labeled points indicate leading edge genes in the GSEA analysis. Grey violins indicate the distribution of FC values within each pathway, and rug plot along x axis indicates the full distribution of FCs estimates for the cough aerosol culture-positive effect. Black curves indicate the running enrichment score deviation from zero along the y axis.

We next used a genome-wide approach to determine whether a specific host inflammatory signature was associated with cough aerosol culture-positive status independent of other disease characteristics in a subset of cough aerosol culture -positive (N = 29) and cough aerosol culture -negative (N = 29) participants (Supplementary Table 2). We measured whole blood RNA-seq profiles from a pre-treatment sample of persons with pulmonary TB and found differentially expressed genes associated with cough aerosol culture -positive status (100 genes with FDR < 0.2). After a stepwise evaluation of covariates and assessment of model fit (based on improvement of > 10% of genes), bacillary load (measured by Ct value) was the only covariate included in the final model. We examined covariate-adjusted expression values and found that no differentially expressed genes (DEGs) were independently associated with cough aerosol culture positivity (Fig. 2A, 2B & Supplemental Fig. 1C). In contrast, many DEGs remained associated with bacterial burden (1129 genes with FDR < 0.2; 40 genes with FDR < 0.05; min FDR = 0.0008, Supplemental Table 3). Together, these data suggest that DEGs are associated with several features of TB presentation but are not independently associated with cough aerosol culture-positive status after adjusting for bacillary burden.

We next used Gene Set Enrichment Analysis (GSEA) to examine whether transcriptional signatures were associated with cough aerosol culture-positive status. Using an adjusted model with bacillary burden as a covariate and assessing for concordant directionality of effect, several gene sets were associated with bacillary load or cough aerosol culture-positive status and represented a diverse set of cellular processes (Fig. 2C). Although there was overlap among cough aerosol culture-positive and bacillary burden associations, the majority of gene sets were associated with concordant directionality with either, but not both, traits (Fig. 2C). We found eight Hallmark gene sets that were associated either exclusively with cough aerosol culture-positive status, or with opposite directionality as bacillary burden. Hallmark_Angiogenesis was associated with cough aerosol culture-positive status while seven gene sets were associated with cough aerosol culture-negative status (Fig. 2C and Supplemental Fig. 4). There were 10 leading-edge genes in the Angiogenesis gene set with functional associations with cellular proliferation, ligand:receptor interactions, and the extracellular matrix. (Fig. 2D) Together, these data suggest that several gene sets were associated with cough aerosol culture-positive status independent of bacillary burden.

## DISCUSSION

The primary findings of TBAIT suggest that host inflammatory signatures are associated with Mtb aerosolization independent of bacillary load and cavitary lung disease. We extend previous studies which determined several non-immunologic host factors that are associated with cough aerosol culture status. (25, 29) We found that higher serum CRP levels, sputum cytokines, and whole blood transcriptional signatures were associated with Mtb aerosolization. These data highlight potential insights into the biology of Mtb transmission events as well as biomarkers to identify highly infectious individuals.

Based on our findings and prior studies, cough aerosol cultures are superior to sputum smear analysis in predicting Mtb transmission events and are likely the best estimators of TB infectiousness currently.(24, 26–28) Although cough aerosol culture-negative patients may transmit Mtb, cough aerosol cultures allow for a more accurate assessment of relative infectiousness than traditional measures of sputum bacillary load. Prior studies found that bacillary burden, mucoid sputum, stronger cough and higher Karnofsky performance score were associated with cough aerosol culture positivity.(25, 29) Theron et al in the largest cough aerosol culture study of TB patients to date(29) found that higher peak cough flow rate, higher bacillary load, lack of HIV infection and lower “TB symptom score” were independent risk factors for cough aerosol culture positivity. In this study, a lower TB symptom score indicated a lower burden of findings attributable to TB disease as it is a summation of points for TB-related symptoms (cough, hemoptysis, dyspnea, chest pain, fever, night sweats), TB-related signs (anemia, tachycardia), lung auscultation findings, and malnutrition (low BMI, low MUAC). Our study adds the novel association between cough aerosol culture-positive status and higher serum CRP levels, an acute phase reactant that is produced in the liver and is a non-specific marker of inflammation. CRP has been extensively evaluated as a triage test for TB, particularly among PLWH,(49) is endorsed by WHO as a TB screen and is available as a point-of-care test.(48) Taken together, these findings suggest common features of a cough aerosol culture-positive phenotype: younger persons with few symptoms (aside from cough), preserved body mass, a higher bacillary burden, and higher systemic inflammation. If accurate, the proposed phenotype of highly infectious persons with TB would likely describe individuals who are younger(16–18, 29), and active rather than moribund(25, 29), increasing the opportunities for TB transmission events. We developed a clinical prediction tool to identify highly infectious persons with TB (cough aerosol culture positive) that could have utility in TB control interventions, for example to inform contact investigations and TB preventive therapy administration, and to improve precision in clinical trials of TB preventive therapy and vaccines. While promising, the risk score that we present requires external validation.

Potential mechanisms underlying increased Mtb aerosolization and transmission include high sputum bacillary loads, Mtb strain features, cough characteristics (e.g., propulsive strength or frequency), and host inflammation. Our results of significantly higher concentrations of sputum CXCL8 and 1L-1β, as well as higher serum CRP, in participants with culturable aerosols support the role of systemic inflammation in TB infectiousness. Sputum cytokines have been evaluated as biomarkers in the diagnosis of TB and treatment monitoring.(50–52) However, to our knowledge no prior studies have evaluated their association with infectiousness measured by culturable aerosols. A balance in the host inflammatory response is needed to prevent dissemination of Mtb (resulting in part from insufficient inflammation) while limiting tissue destruction and other complications from excessively robust inflammatory responses.(53, 54) The stimulation of inflammatory pathways that regulate Mtb aerosolization and transmission may exert an additional evolutionary pressure on the immune response to Mtb. To explore potential mechanisms of Mtb aerosolization, we discovered a whole blood transcriptomic signature associated with cough aerosol culture -positivity after adjustments for bacillary load. Given the timing of sample collection at the time of diagnosis, we cannot distinguish whether index case inflammatory pathways cause Mtb aerosolization or vice versa. If the inflammatory pathways associated with Mtb aerosolization are regulated by immunogenetically encoded mechanisms, then the Angiogenesis signaling pathway, which was enriched in cough aerosol culture-positive participants may offer causal insights. Of the 10 leading edge genes differentiating cough aerosol culture-positive versus -negative status, several are involved in the extracellular matrix (COL5A2, FGFR1, ITGAV) and cell development and proliferation (JAG1, PDGFA, FSTL1) pathways which offer potential insights into aerosolization mechanisms. For example, extracellular molecules secreted by these pathways could provide an alternative milieu surrounding the bacterium which modulates the surface structure or metabolic state of Mtb and its subsequent capacity to transmit. Previous work in the *M. marinum* zebrafish model uncovered an important role for vascularization and angiogenesis in granuloma formation and control of bacterial dissemination.(55, 56) Enzymatically modified trehalose dimycolate (TDM), an immunogenic cell wall lipid, induced angiogenesis through a VEGF pathway. These studies highlight potential mechanisms that could influence Mtb aerosolization via modulation of angiogenesis-dependent inflammatory pathways. Similarly, cytokine pathways (e.g., Interferon Alpha Response and Interferon Gamma Response gene sets enriched in cough aerosol culture -negative individuals) from activated immune cells could modulate Mtb’s state and survival before, during, and after propulsion from the host airway. Regardless of the causal pathway, the inflammatory profiles provide a potential biomarker of infectiousness that could rapidly identify Mtb superspreaders.

Our study supports prior findings of significant individual host variation in infectiousness among patients with TB and suggests that factors beyond bacillary burden determine infectiousness.(19, 21, 22, 25, 26, 57) While CASS, modeled on cough aerosol production, is the best-studied method for assessing infectiousness, recent studies have drawn attention to detectable Mtb from non-cough respiratory maneuvers. (58, 59) (32, 35) Williams and colleagues demonstrated that when persons with confirmed and suspected TB wore face masks with collection strips (face mask sampling or FMS) while breathing normally, Mtb DNA was detected in up to 90% of persons. (58, 59) The authors subsequently found a modest association between FMS detection of Mtb and increased TB infection in close contacts.(60) RASC is a device to capture bioaerosols that are then evaluated for viable Mtb using a fluorescent trehalose analogue.(32, 35) In a study of 38 GeneXpert positive participants who performed respiratory maneuvers (tidal breathing, voluntary cough, forced vital capacity) while seated in the RASC, 88% of participants produced at least one sample positive for Mtb and all three maneuvers were equally likely to produce viable Mtb.(35) Further investigations are needed to determine differences in findings from CASS, FMS, and RASC, especially as they relate to actual transmission events.

Our study has several limitations. First, we did not evaluate whether Mtb microbiologic factors are associated with cough aerosol culture status. A previous investigation of Mtb genetic variants and cough aerosol culture positivity did not demonstrate associations.(29) Second, our transcriptomic data derives from whole blood which precludes cell-specific insights. Despite this limitation, pathway analysis suggests possible cellular sources of mechanisms which can be tested in future studies. Third, we were unable to assess causality of immunologic pathways associated with cough aerosol culture positivity which may precede Mtb aerosolization or be a consequence of it. However, we did evaluate for possible confounding and adjusted for bacillary load to identify gene sets that are independently associated with cough aerosol culture status. Finally, we enrolled participants during the COVID-19 pandemic and the impact of SARC-CoV-2 on CRP level, transcriptional profiles or other study variables is not known. There were also several strengths to our study. First, all participants underwent CASS procedures prior to the initiation of anti-tuberculosis therapy, which is known to rapidly impact cough aerosol culture results.(29, 61) Second, we adhered to a rigorous definition of cough aerosol culture status in which we excluded participants who had more than two CASS plates contaminated with overgrowth. Third, we demonstrated that cough aerosol culture-positive status was strongly associated with evidence of TB transmission in household contacts based on QFT results.

Our findings, along with those from prior studies, suggest that host characteristics and biomarkers may identify the most infectious patients with TB. CRP, already recommended as a screening test for TB by the World Health Organization, may have a role in identifying highly infectious persons with TB.(62, 63) While persons with pulmonary TB who are cough aerosol culture-negative patients may transmit Mtb, identifying the most infectious persons would allow targeted treatment interventions (such as directly observed therapy) and focused contact investigations in home and community sites with provision of TB preventive therapy. Further elucidation of inflammatory transcriptional signatures associated with TB infectiousness are important not only for understanding mechanisms and developing new therapies, but also for the possibility of developing a diagnostic tool to identify individuals who are the most infectious.

## Figures and Tables

**Table 1:** Characteristics of Participants with TB (Index participants) overall and by cough aerosol culture (CAC) status.

Characteristics	Total n = 142	CAC+ n = 43	CAC− n = 99	Odds Ratio	p-value
Age (med, IQR)	35 (27, 44)	32 (24, 39)	36 (28, 48)	0.95 (0.91–0.98)	0.005
Female	39 (27%)	8 (19%)	31 (31%)	0.50 (0.21–1.21)	0.12
BMI (n = 141)	19 (17, 21)	19 (18, 21)	19 (17, 21)	1.02 (0.92–1.14)	0.69
MUAC*, cm (n = 141)	23 (21, 25)	24 (21, 26)	22 (21, 25)	1.14 (1.00–1.30)	0.04
PLWH**	14 (10%)	4 (9%)	10 (10%)	0.91 (0.27–3.09)	0.88
Taking ART?	7 (50%)	4 (100%)	3 (30%)		0.02
CD4 T-cell count (n = 13)	193 (67, 231)	218 (105, 397)	178 (67, 216)	1.00 (1.00–1.01)	0.81
Prior hx of TB (n = 140)	27 (19%)	8 (19%)	19 (20%)	0.94 (0.37–2.35)	0.89
Enrolled thru Prevalence Survey	26 (18%)	0	26 (26%)		
Cavitary CXR	99 (70%)	38 (88%)	61 (62%)	4.73 (1.71–13.08)	0.003
CXR quadrants (n = 140)				1.50 (1.02–2.20)	0.04
0	1 (1%)	0	1 (1%)		
1	47 (34%)	8 (19%)	39 (40%)		
2	53 (38%)	18 (42%)	35 (36%)		
3	28 (20%)	15 (35%)	13 (13%)		
4	11 (8%)	2 (5%)	9 (9%)		
Cough (n = 141)	133 (95%)	43 (100%)	91 (93%)		0.07 (chi2)
Fever (n = 141)	92 (65%)	35 (81%)	57 (58%)	3.15 (1.32–7.49)	0.1
Weight loss (n = 141)	120 (85%)	41 (95%)	79 (81%)	1.00 (0.97–1.03)	0.76
Night sweats	108 (77%)	36 (84%)	72 (73%)	1.86 (0.74–4.69)	0.19
Cough duration (days) (n = 134)	8 (4,12)	8 (4, 12)	6 (4, 12)	1.03 ( 1.0–1.07)	0.1
LCQ Score*** (n = 140)	14.5 (12.2, 16.7)	13.1 (10.4, 14.4)	15.5 (12.8, 17.2)	0.81 (0.71–0.92)	0.001
CPF^†^, mL	260 (170, 370)	225 (170, 350)	270 (170, 370)	1.00 (1.0–1.0)	0.23
Current tobacco use	32 (23%)	9 (21%)	23 (23%)	0.86 (0.36–2.06)	0.74
Xpert Ct	18.8 (16.6, 23.3)	16.4 (14.6, 18.1)	19.7 (17.8, 25.1)	0.73 (0.63–0.84)	<0.001
Xpert Grade				2.12 (1.35–3.22)	0.001
Trace	4 (3%)	0	4 (4%)		
Very Low	9 (6%)	1 (2%)	8 (8%)		
Low	28 (20%)	3 (7%)	25 (26%)		
Medium	45 (32%)	15 (35%)	30 (30%)		
High	56 (39%)	24 (56%)	32 (32%)		
Sputum Smear Grade				1.62 (1.16–2.26)	0.004
Negative	15 (11%)	1 (2%)	14 (14%)		
Scanty	9 (6%)	2 (5%)	7 (7%)		
1+	33 (23%)	5 (12%)	28 (28%)		
2+	35 (25%)	16 (37%)	19 (19%)		
3+	50 (35%)	19 (44%)	31 (31%)		
TTD^††^ (days) (n = 136)	5 (4, 9)	4 (3, 5)	6 (4, 10)	0.75 (0.63–0.88)	<0.001
Sputum Appearance					
Thin	46 (32%)	10 (23%)	36 (36%)	REF	
Thick	91 (64%)	32 (74%)	59 (60%)	1.95 (0.86–4.44)	0.11
Bloody	5 (4%)	1 (2%)	4 (4%)	0.90 (0.09–8.98)	0.93
CRP (mg/dL) (n = 141)	67 (26, 96)	88 (57, 131)	51 (15, 86)	1.02 (1.01–1.03)	<0.001
WBC	7.1 (5.7, 9.1)	7.7 (6.4, 10.1)	6.8 (5.6, 8.6)	1.19 (1.03–1.36)	0.02
HbA1C (n = 141)	5.7 (5.3, 6.0)	5.8 (5.5, 6.1)	5.6 (5.1, 6.0)	1.1 (0.79–1.45)	0.67
Hgb	13 (11.9, 14.2)	13.0 (12.0, 13.9)	13.1 (11.8, 14.3)	1.00 (0.84–1.19)	1

* MUAC, Mid-upper arm circumference;

** PLWH, person living with HIV;

*** LCQ, Leicester Cough Questionnaire;

† CPF, Cough peak flow;

†† TTD, Time to detection of Mtb growth in liquid media

**Table 2:** Household Contact Characteristics overall and by index cough aerosol culture (CAC) status

Characteristics	Total n = 143	CAC+n = 27	CAC− n = 116	OR	p-value
**Household Contact Characteristics**					
Age (med, IQR)	13 (5, 28)	12 (5, 28)	14 (5, 29)	0.99 (0.96–1.02)	0.56
Female	76 (53%)	15 (56%)	61 (53%)	1.13 (0.492.62)	0.78
BMI* (n = 136)	20 (17, 24)	19 (15, 25)	20 (17, 24)	0.97 (0.911.04)	0.36
MUAC**, cm (n = 137)	22 (16.0, 25.5)	19.2 (15.5, 29.5)	22 (16.3, 25.5)	1.00 (0.941.07)	0.93
PLWH^†^ (n = 112)	5 (4%)	1 (5%)	4 (4%)	1.24 (0.1311.72)	0.85
**Index Characteristics**					
Age (n = 141)	34 (26, 44)	37 (29, 41)	32 (25,47)	1.00 (0.971.02)	0.73
PLWH^†^ (n = 141)	17 (12%)	2 (7%)	15 (13%)	0.53 (0.112.46)	0.42
BMI* (n = 138)	19 (18, 23)	18 (18, 21)	19 (18, 23)	1.07 (0.971.18)	0.18
MUAC* (n = 138)	23.4 (20.8, 25.7)	22.9 (22.4, 25.5)	23.5 (20.8, 25.8)	1.05 (0.931.19)	0.42
Xpert Ct (n = 141)	20.5 (17.6, 27.1)	19.0 (16.6, 20.4)	22.7 (17.8, 27.3)	0.80 (0.710.90)	<0.001
Xpert Grade (n = 141)				2.15 (1.283.59)	0.004
Trace	7 (5%)	0	7 (6%)		
Very Low	17 (12%)	0	17 (15%)		
Low	31 (22%)	2 (7%)	29 (25%)		
Medium	59 (42%)	19 (70%)	40 (35%)		
High	27 (19%)	6 (22%)	21 (18%)		
Smear Grade (n = 141)				1.08 (0.801.48)	0.61
Negative	27 (19%)	4 (15%)	23 (20%)		
Scanty	6 (4%)	1 (4%)	5 (4%)		
1+	42 (30%)	7 (26%)	35 (31%)		
2+	32 (23%)	10 (37%)	22 (19%)		
3+	34 (24%)	5 (19%)	29 (25%)		
TTD^††^ (days) (n = 130)	6 (4, 12)	5 (4, 6)	7 (4, 12)	0.86 (0.750.98)	0.02
Cavitary CXR (n = 141)	78 (55%)	24 (89%)	54 (47%)	8.89 (2.5331.19)	0.001
CRP (n = 136)	46 (9, 75)	51 (26, 96)	46 (6, 74)	1.01 (1.001.02)	0.06
CXR quadrants (n = 134)				1.74 (1.052.86)	0.03
0	0	0	0		
1	53 (40%)	6 (22%)	47 (44%)		
2	55 (41%)	11 (41%)	44 (41%)		
3	21 (16%)	10 (37%)	11 (10%)		
4	5 (4%)	0	5 (5%)		
Number of contacts per index					0.01
1	19	4	15		
2	11	3	8		
3	9	1	8		
4	8	1	7		
5	5	2	3		
6	3	0	3		
**QuantiFERON Result in HHC (n = 127)**					
QFT-neg	49 (39%)	3 (11%)	46 (46%)		0.005
QFT-pos	76 (60%)	22 (85%)	55 (53%)		
Indeterminate	2 (2%)	1 (4%)	1 (1%)		
Mean IFN-γ (lU/mL) (SD) (indeterminate excluded)	2.27 (2.54)	4.02 (2.94)	1.82 (2.22)		<0.001
Median IFN-γ (IU/mL) (IQR) (indeterminate excluded)	1.01 (0.02–4.38)	4.25 (0.90–5.91)	0.71 (0.01–3.56)		
QFT-positive HHCs, Mean IFN-γ (lU/mL) (SD) (n = 76)	3.76 (2.28)	4.76 (2.58)	3.35 (2.04)		0.01
QFT-positive HHCs, Median IFN-γ (IU/mL) (IQR) (n = 76)	3.71 (1.825.02)	4.59 (3.356.97)	3.46 (1.784.71)		

* BMI, Body mass index;

** MUAC, Mid-Upper Arm Circumference;

† PLWH, person living with HIV;

†† TTD = Time to detection of Mtb growth in liquid media

**Table 3:** Mixed methods bivariate & multivariable analyses of index and household contact characteristics associated with QFT result in household contacts (excluding indeterminate results)

Predictors^†^	OR	95% CI	p-value	aOR	95% CI	p-value
**Household Contact Characteristics**						
Age	1.04	1.01–1.08	0.046	1.04	1.00–1.07	0.04
Female	1.00	0.41–2.47	0.99			
PLWH*	2.97	0.25–35.32	0.39			
**Index Characteristics**						
Cough aerosol culture positive	8.68	1.62–46.60	0.01	9.37	1.39–63.12	0.02
Age	1.03	0.99–1.07	0.15			
PLWH*	0.05	0.01–0.46	0.008	0.06	0.01–0.58	0.02
Xpert Ct	0.93	0.84–1.02	0.13			
Xpert semi-quantitative grade	1.25	0.84–1.87	0.27			
TTD** (days)	0.88	0.73–1.05	0.16			
AFB-smear grade†	1.45	0.95–2.21	0.09			
CRP level (mg/dL)	1.01	1.00–1.03	0.09			
Cavitary chest X-ray	3.37	1.09–10.44	0.04			
CXR Quadrants	1.58	0.78–3.20	0.21			
Body Mass Index	0.97	0.84–1.13	0.70			
MUAC^†^	0.93	0.78–1.11	0.41			

* PLWH, person living with HIV;

** TTD, Time to detection of Mtb growth in liquid media;

† MUAC, Mid-Upper Arm Circumference

† Predictors were tested in forward selection stepwise multivariable models and compared with likelihood ratio test. Best performing model with adjusted odds ratios shown in final three columns.

**Table 4:** Multivariable model of index characteristics associated with cough aerosol culture positivity

Characteristics	OR	95% CI	p-value	aOR	95% CI	p-value
MUAC^†^ (cm)	1.14	1.00–1.30	0.04	1.42	1.17–1.73	<0.001
CRP level (mg/dL)	1.02	1.01–1.03	<0.001	1.02	1.01–1.03	0.001
Xpert Ct	0.73	0.63–0.84	<0.001	0.76	0.64–0.90	0.002
Age (years)	0.95	0.91–0.98	0.005	0.94	0.90–0.99	0.02
TTD** (days)	0.75	0.63–0.88	<0.001	0.80	0.64–1.00	0.047
Female	0.50	0.21–1.21	0.12			
BMI	1.02	0.92–1.14	0.69			
Sputum smear grade	1.62	1.16–2.26	0.005			
Xpert semi-quantitative grade	2.12	1.35–3.22	0.001			
PLWH†	0.91	0.27–3.09	0.88			
Fever	3.15	1.32–7.49	0.10			
Cough Duration	1.03	1.00–1.07	0.10			
LCQ^††^ Score	0.81	0.71–0.92	0.001			
CPF***	1.00	1.00–1.00	0.23			
Cavitary Chest X-ray	4.73	1.71–13.08	0.003			
Chest x-ray quadrants	1.50	1.02–2.20	0.04			
WBC	1.19	1.03–1.36	0.02			

† MUAC, Mid-Upper Arm Circumference;

** TTD, Time to detection of Mtb growth in liquid media;

† PLWH, Person living with HIV;

†† LCQ, Leicester Cough Questionnaire;

*** CPF, Cough peak flow
